# Effective primary care management of type 2 diabetes for indigenous populations: A systematic review

**DOI:** 10.1371/journal.pone.0276396

**Published:** 2022-11-10

**Authors:** Sahil Chopra, Tahne Joseph Lahiff, Richard Franklin, Alex Brown, Roy Rasalam

**Affiliations:** 1 Princess Alexandra Hospital, Brisbane, QLD, Australia; 2 University of Queensland, Brisbane, QLD, Australia; 3 Royal Brisbane and Women’s Hospital, Brisbane, QLD, Australia; 4 College of Public Health, Medical and Vet Sciences, James Cook University, Townsville, QLD, Australia; 5 Indigenous Genomics, Australian National University and Telethon Kids Institute, Nedlands, Australia; 6 Public Health Medicine, Queensland Health, Townsville, QLD, Australia; University of South Australia, AUSTRALIA

## Abstract

**Background:**

Indigenous peoples in high income countries are disproportionately affected by Type 2 Diabetes. Socioeconomic disadvantages and inadequate access to appropriate healthcare are important contributors.

**Objectives:**

This systematic review investigates effective designs of primary care management of Type 2 Diabetes for Indigenous adults in Australia, Canada, New Zealand, and the United States. Primary outcome was change in mean glycated haemoglobin. Secondary outcomes were diabetes-related hospital admission rates, treatment compliance, and change in weight or Body Mass Index.

**Methods:**

Included studies were critically appraised using Joanna Briggs Institute appraisal checklists. A mixed-method systematic review was undertaken. Quantitative findings were compared by narrative synthesis, meta-aggregation of qualitative factors was performed.

**Results:**

Seven studies were included. Three reported statistically significant reductions in means HbA1c following their intervention. Seven components of effective interventions were identified. These were: a need to reduce health system barriers to facilitate access to primary care (which the other six components work towards), an essential role for Indigenous community consultation in intervention planning and implementation, a need for primary care programs to account for and adapt to changes with time in barriers to primary care posed by the health system and community members, the key role of community-based health workers, Indigenous empowerment to facilitate community and self-management, benefit of short-intensive programs, and benefit of group-based programs.

**Conclusions:**

This study synthesises a decade of data from communities with a high burden of Type 2 Diabetes and limited research regarding health system approaches to improve diabetes-related outcomes. Policymakers should consider applying the seven identified components of effective primary care interventions when designing primary care approaches to mitigate the impact of Type 2 Diabetes in Indigenous populations. More robust and culturally appropriate studies of Type 2 Diabetes management in Indigenous groups are needed.

**Trail registration:**

Registered with PROSPERO (02/04/2021: CRD42021240098).

## Introduction

Indigenous peoples of Australia, Canada, New Zealand (NZ), and the United States (US) are culturally, spiritually, geographically, and racially, distinct people. However, these populations all live within countries with developed economies and share common experiences of colonisation, marginalisation, and land rights disputes which perpetuated cycles of socioeconomic disadvantage and unequal power relationships with the institutions of civil society [1, 2]. Disadvantages in social determinants of health establish vicious cycles of poor healthcare access, perpetuating poor health outcomes [1–5]. The global burden of T2DM disproportionately affects First Nations People [6]. Globally, 9.3% of the population lives with diabetes; this is predicted to increase to 10.9% by 2045 [7]. In the US, American Indian/Alaska Native (AI/AN) groups have the highest prevalence of diabetes by ethnicity (14.7% vs 9.4% for US overall), with some communities experiencing prevalence rates over 50% [8, 9]. In Australia, Aboriginal and Torres Strait Islander groups have 7.9% prevalence of diabetes (4.9% Australia overall) [10]. However, this is based on self-reported data and a prevalence study of diabetes concluded only 81% of Indigenous Australians with diabetes are diagnosed [11].

Gibson et al.’s 2015 systematic review assessed the impact of primary care attributes on health outcomes of First Nations People living with diabetes in literature published till March 2011 [12]. Due to a small number of predominantly observational studies forming the evidence base and a reliance on quantitative analysis alone, it concluded evidence was inadequate to inform policy-relevant decisions about optimal primary care design and delivery [12]. Since 2011, diabetes has continued to disproportionately affect Indigenous peoples at an increasing rate [2, 3, 6–8]. Policies addressing underlying social determinants of health may modify access to and receipt of evidence-based healthcare services and resultant health outcomes [1–5]. Yet, these factors are profoundly difficult to modify, particularly in the context of historical marginalisation and institutional racism [1–5]. Multi-faceted approaches are required to achieve this [5]. Alternative primary care models include primary care delivery in non-health centre settings, specialist outreach delivery in primary care settings, financial incentives for staff or patients or both, telecommunications strategies, quality improvement programs, and care coordination or case management. This systematic review analyses primary care interventions for T2DM management in Indigenous populations to describe characteristics in interventions associated with favourable biomarker or community responses or both.

### Why primary care?

As diabetes is a chronic disease, effective management requires patient education and agency to enable engagement with self-management, regular monitoring, lifestyle modifications, and pharmacological management [13]. This requires the health system to deliver care in ways accepted by and accessible for its patients. Primary care is healthcare provided in the community and often acts as the coordination centre for a patient’s holistic healthcare needs. This is particularly relevant in the context of Aboriginal Community Controlled Health Organisations (ACCHOs), which were established for that very purpose [14]. Similarly, Alaska Native tribal health organisations, owned by Alaska Native and American Indian tribes, provide primary care to customer owners [15]. As such, primary care is the ideal setting for effective diabetes management and the prevention of complications [16].

### International primary care systems

The 4 countries of interest have varying primary healthcare systems with subsidised services available for First Nations People. The US operates a private health system with some government-owned medical facilities. Native Americans from federally recognised tribes are eligible for direct medical care through the Indian Health Service (IHS), an operating division of the Department of Health and Human Services. Eligible Native Americans also receive IHS coverage if referred to non-IHS health services. Approximately two thirds of American Indians and Alaska Natives receive care through the IHS [17]. In Canada, under the Canada Health Act 1984, through Medicare there are no out-of-pocket primary care costs for First Nations People. First Nations People can access either Community Health Centres or Aboriginal Health Access Centres for primary care. Costs for additional necessary services are covered through the Non-Insured Health Benefits Scheme [18].

In Australia, Indigenous populations can utilise primary care at Community Controlled Health Organisations formed from partnerships between the Australian Government and local Indigenous Non-Governmental Organisations [19]. Alternatively, mainstream primary health centres, accessible to the rest of the population, can also provide primary care to Indigenous Australians. Through the Closing the Gap campaign and National Diabetes Services Scheme, there should be no out-of-pocket costs for Indigenous Australians for primary care services [20]. New Zealand has a Māori Health Strategy–He Korowai Oranga [21]. In NZ, Māori residents, like non-Māori residents, pay a small gap fee for primary care. However, a new Māori Health Authority has been announced in 2021 to help achieve Māori health goals and introduce more comprehensive access models [22].

### Barriers to primary care for First Nations People

Despite the various incentives and primary care models trialled in high income countries to improve health outcomes for First Nations People [1, 20, 23, 24], many barriers prevent adequate access to primary care [25]. The health system and health researchers are not a passive recipient waiting for Indigenous people to engage with interventions. Barriers arise from the western health system itself, such as the individualistic nature of the system, centre-based rather than home-based delivery, and inadequate staff cultural education leading to stereotyping and assumptions [15, 23, 25]. Individual level barriers against primary care access for Indigenous people can include income-related factors, such as transport and affordability, and distrust in government agencies and power imbalances in interactions between healthcare providers and First Nations People extending from colonisation and past discriminatory policies [1–5, 25]. Overcoming these barriers is equally about the motivation of parties in the health system to actively provide accessible, culturally-safe care as it is about motivating and engaging Indigenous people to engage with the health system. The purpose of this systematic review is to highlight a range of evidence-based primary care strategies to overcome such barriers and facilitate utilisation of appropriate health services by First Nations People to improve T2DM outcomes.

### Aims

This study, rather than describing the already-defined value of primary care for the long-term management of diabetes, aims to determine which designs of primary care interventions are effective in the management of T2DM in Indigenous adult populations in high income countries to generate recommendations for practice and policymakers. Our chosen population is Indigenous adults (aged 18 and over) in Australia, Canada, NZ, and the US with T2DM. Interventions explored are active modifications of primary care by health practitioners to reduce barriers to access for Indigenous patient engagement. The primary outcome is change in mean glycated haemoglobin A1c (HbA1c), a marker of glycaemic control over the preceding three months. Secondary outcomes include diabetes-related hospital admission rate, weight and/or Body Mass Index (BMI) change, and adherence to diabetes treatment. RCT, cluster-control studies, before-and after-studies, and observational studies conducted in Australia, Canada, NZ, or the US were accepted.

## Methods

### Sources

An electronic search for peer-reviewed and grey literature publications in English for the period 2011–2020 was undertaken. The project was registered with The International Prospective Register of Systematic Reviews (PROSPERO) on April 2, 2021 (CRD42021240098). The search strategy was created in consultation with a specialist university librarian. S1 Table shows the extensive list of keywords. Cumulative Index to Nursing and Allied Health Literature (CINAHL) and Medline key terms were strung together in three streams combined with ‘AND’ commands. The first stream pertained to primary care interventions, the second contained synonyms for T2DM, and the third described included Indigenous populations. The combined search was run in CINAHL Complete, the Cochrane Library, Emcare on Ovid, Medline, and Scopus databases. For completeness, co-author AB was consulted to ensure grey literature coverage—no further articles or reports were deemed missing after searching Health Canada, Australian Institute of Health and Welfare, The George Institute, Ministry of Health NZ, and Centers for Disease Control and Prevention (CDC) Reports.

**Fig 1:**
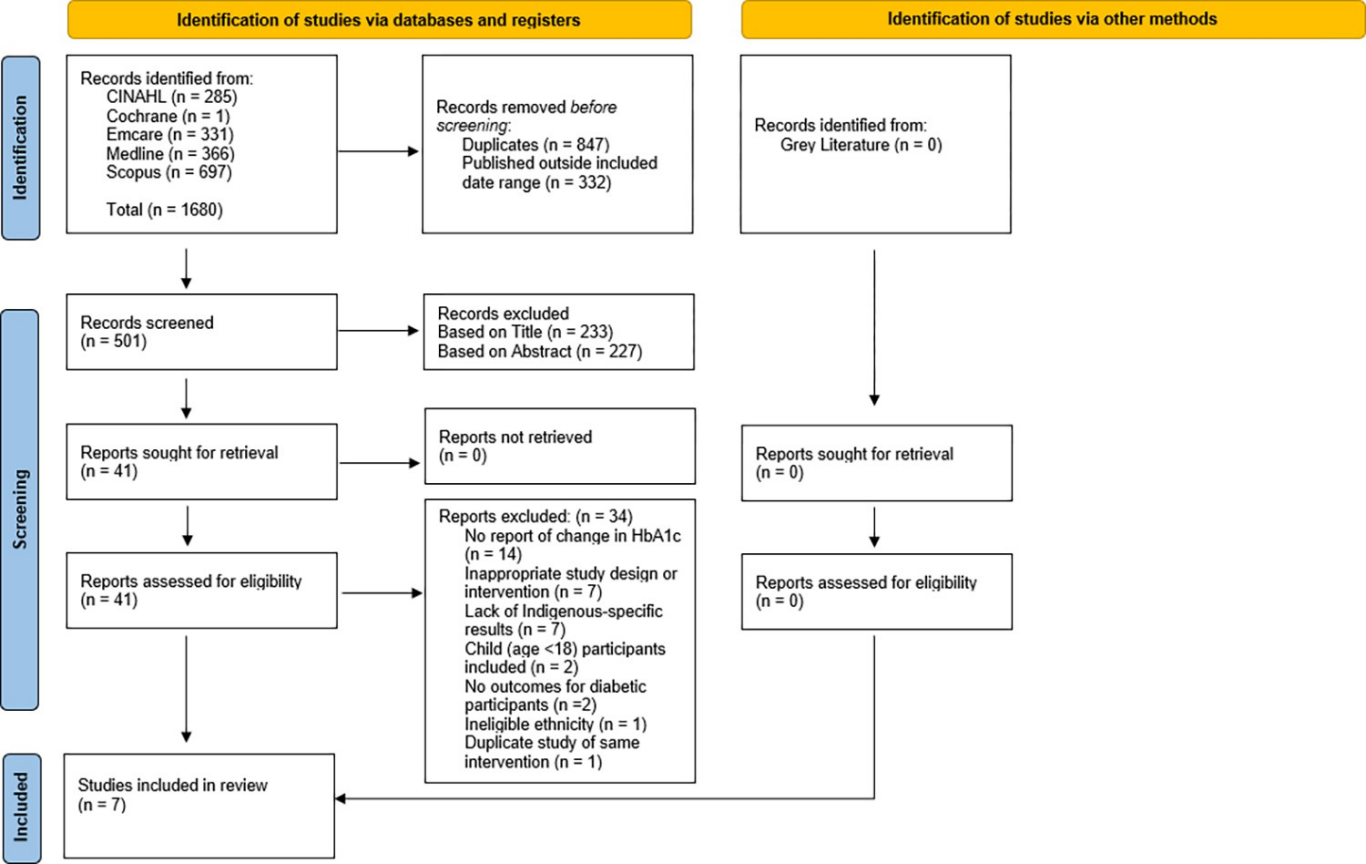
PRISMA flowchart of included studies.

Findings are presented in the Preferred Reporting Items for Systematic Revies and Meta-Analyses (PRISMA) Flowchart (Fig 1) [26]. The search results were imported into an Endnote™ X9 database, where studies published outside the included date range (January 1 2011 to December 31 2020) and duplicates were removed. Title, abstract, and full-text of remaining records were screened independently by two reviewers (SC and TL) for eligibility against inclusion and exclusion criteria. Reasons for exclusion were noted at full-text stage. There were no non-retrievable articles at abstract screening. Disagreements about inclusion and exclusion of articles were resolved by third independent reviewer (RR). The included studies and their characteristics were summarised in Table 1.

**Table 1:** JBI critical appraisal scores [27].

JBI Critical Appraisal Tool Scores														
**Randomised Controlled Trials**	Q1	Q2	Q3	Q4	Q5	Q6	Q7	Q8	Q9	Q10	Q11	Q12	Q13	**Overall**
McDermott et al.—Community health workers improve diabetes care in remote Australian indigenous communities: Results of a pragmatic cluster randomized controlled trial (2015)														**Good (9/13)**
Canuto et al.—Pragmatic randomised trial of a 12-week exercise and nutrition program for Aboriginal and Torres Strait Islander women: Clinical results immediate post and 3 months follow-up (2012)														**Good (8.5/13)**
**Cohort Studies**	Q1	Q2	Q3	Q4	Q5	Q6	Q7	Q8	Q9	Q10	Q11			**Overall**
Smith et al.—Analysis of a primary care led diabetes annual review programme in a multi ethnic cohort in Wellington, New Zealand (2011)														**Moderate (6/11)**
Titchener—A patient-centred clinical approach to diabetes care assists long-term reduction in HbA1c (2014)														**Poor (4.5/11)**
Pratte et al.—Recruitment and effectiveness by cohort in a case management intervention among American Indians and Alaska Natives with diabetes (2019)														**Good (8/11)**
**Quasi-Experimental Study**	Q1	Q2	Q3	Q4	Q5	Q6	Q7	Q8	Q9					**Overall**
Shah et al.—A home-based educational intervention improves patient activation measures and diabetes health indicators among Zuni Indians (2015)														**Moderate (5/9)**
Wilken et al.—Talking Circles to Improve Diabetes Self-care Management (2017)														**Moderate (5.5/9)**

Green = Yes (Score 1); Yellow = Unsure (Score 0.5), Red = No (Score 0).

Overall: >70% = Good; 70–50% = Moderate; <50% = Poor.

Refer to S1 Fig for list of questions.

### Study selection

Eligible studies were English language RCT, cluster-control studies, before- and after- studies, and observational studies conducted in Australia, Canada, NZ, or the US. Studies had to report a primary care intervention, model of care, or service with outcomes reported separately for Indigenous people aged 18 and above with T2DM. Studies were only included if HbA1c was reported; any secondary outcomes could be reported. Only studies published from January 1, 2011, to December 31, 2020, were included, enabling synthesis of a decade of evidence. Studies with less than 10 Indigenous participants were excluded. Qualitative studies and reports solely regarding type 1 diabetes or gestational diabetes were also excluded.

### Rationale

The primary outcome, HbA1c, measures average blood sugar concentration over the previous two-three months. It is the most widely accepted indicator of long-term glycaemic exposure and accepted as the standard measure of diabetes management [28, 29]. This standardised measure allows comparison of interventions and associated outcomes. HbA1c has been demonstrated as a suitable surrogate of risk of complications including retinopathy, nephropathy, neuropathy, cardiovascular risk, and death [29].

The secondary outcomes allow more holistic evaluation of diabetes management efficacy and acceptability. Diabetes-related complications (measured by diabetes-related hospitalisation rate) are directly correlated with poor blood sugar control [29]. Moreover, treatment adherence is essential for effective management of T2DM [30]. Due to inconsistency in outcome reporting across included studies, adherence to diabetes medications, check-ups, and follow-up for trials were included as indicators for adherence to diabetes treatment. These behavioural phenomena demonstrate participant engagement with the primary care intervention, which indicates both increased health practitioner motivation to provide care accepted by Indigenous patients and Indigenous participant motivation to engage with the intervention. Elevated body weight/BMI is a key component of the pathophysiology of insulin resistance in T2DM, and therefore weight reduction is a key goal of effective T2DM management [31]. Due to the variability in reporting of body mass among studies, both body weight and BMI were accepted for this secondary outcome.

As reported in Gibson et al.’s systematic review (2015), there are significant barriers to and lack of research in Indigenous populations, with well-conducted RCT studies being limited [12]. Therefore, observational studies were included in the systematic review to enable sufficient data to stimulate analysis of successful features of primary care interventions for the management of T2DM in the selected populations. Furthermore, our study is unique, as a mixed-method qualitative analysis of included studies was undertaken, rather than relying solely on quantitative data, to obtain reliable findings of effective primary care features to translate into policy and practice recommendations.

### Data extraction

To address reviewer and typographical errors, two reviewers (SC and TL) independently extracted data from eligible studies into an interactive Google Sheet™. A master copy of data matched by both reviewers was inserted into Microsoft Excel™ spread sheets. Column headings were title; author; publication year; years covered in study; study design; study location; indigenous population; age group in study; study quality; primary care intervention; control description (if applicable); number of Indigenous participants; number in intervention, control, total; impact on mean HbA1c; impact on diabetes-related hospital admission rate; impact on treatment adherence; impact on mean weight/BMI. The spread sheet included drop-down data lists for study population and study design. Columns were approved by all five authors. The two sets of data were compared. Data most accurately reflecting each study’s results were included (as agreed by SC and TL, confirmed by RR where discrepancies).

### Assessment of study quality

Included studies were critically appraised independently by two reviewers (SC and TL) using Joanna Briggs Institute (JBI) critical appraisal checklists [27]. The two reviewers used pre-agreed cut-offs for study quality labelling: >70% = good; 50–70% = moderate; <50% = poor. Disagreements were resolved by a third independent reviewer (RR). Critical appraisal results are found in Table 1 and a summary in Table. S1 Fig shows the critical appraisal tools.

### Assessment of heterogeneity

Despite HbA1c being reported in all included studies, study samples being similar across studies, and standardised measuring of outcomes, due to heterogeneity in interventions, single summary measures were not feasible. Our method enabled appreciation of qualitative intervention factors associated with quantitative HbA1c improvements and enhanced engagement with primary care, which in turn may lead to improved clinical outcomes.

### Statistical methods

Due to heterogeneity of HbA1c reporting, no statistical augmentation of HbA1c was performed and impact on HbA1c was directly compared across studies using narrative synthesis [32, 33]. Moreover, the aim of the study was not to demonstrate whether primary care is effective in treating diabetes, but rather to determine features of primary care interventions associated with clinical and behavioural improvements in diabetes care. Hence, a mixed-method systematic review with convergent segregated approach was undertaken as per the JBI Manual for evidence synthesis. Under this approach, a narrative synthesis of quantitative data and meta-aggregation of qualitative data was performed [32, 33].

**Fig 2:**
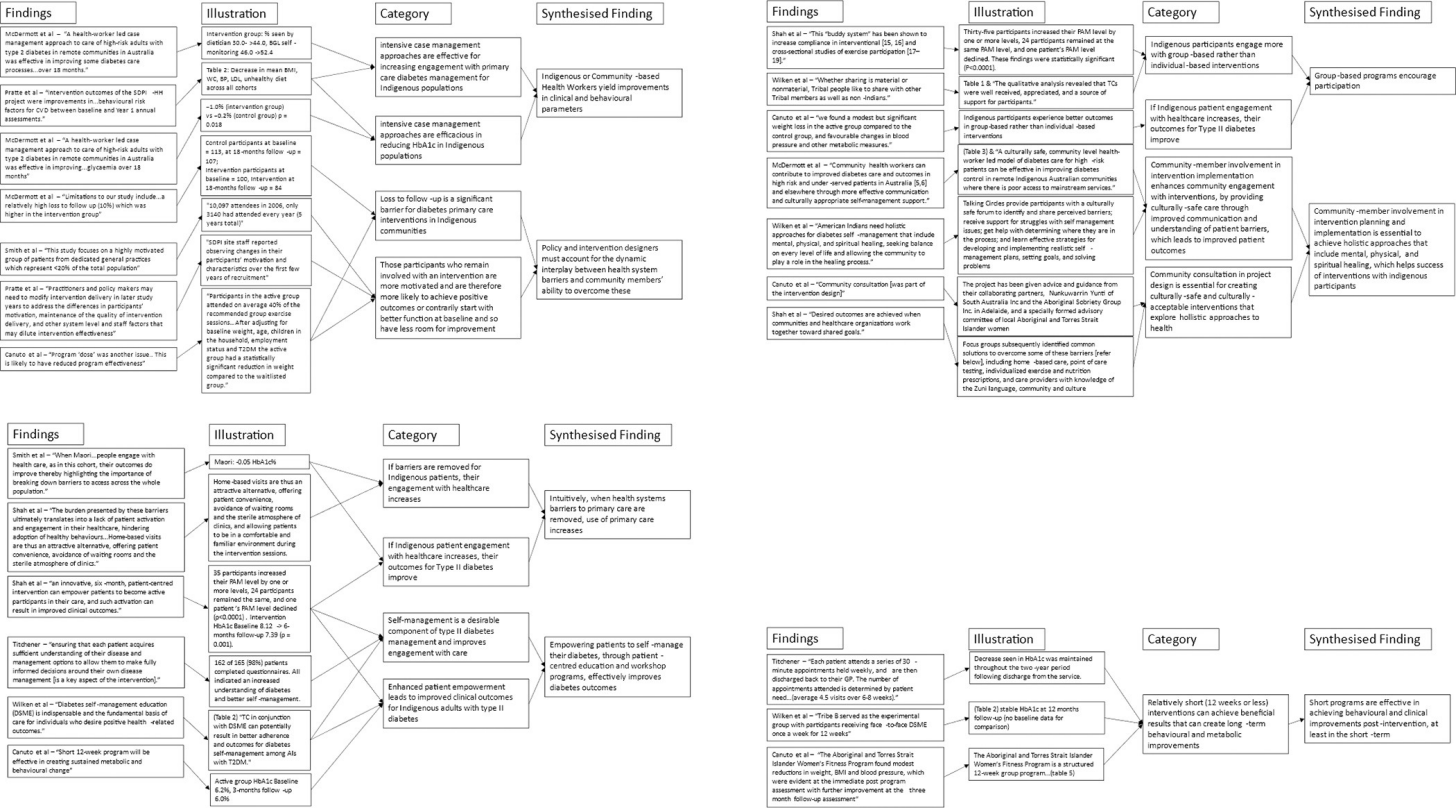
Flowchart of meta-aggregation part 1.

**Fig 3:**
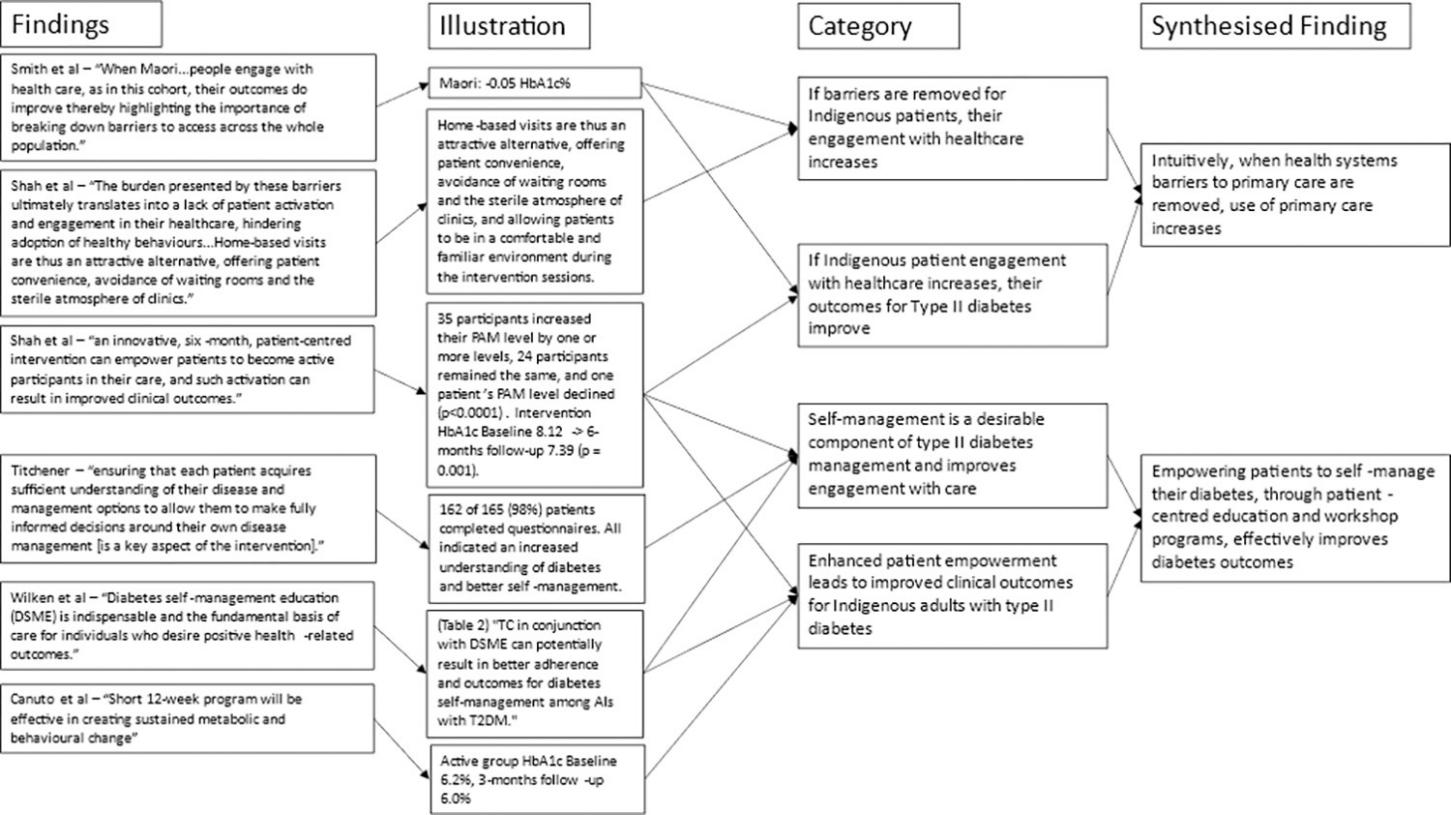
Flowchart of meta-aggregation part 2.

**Fig 4:**
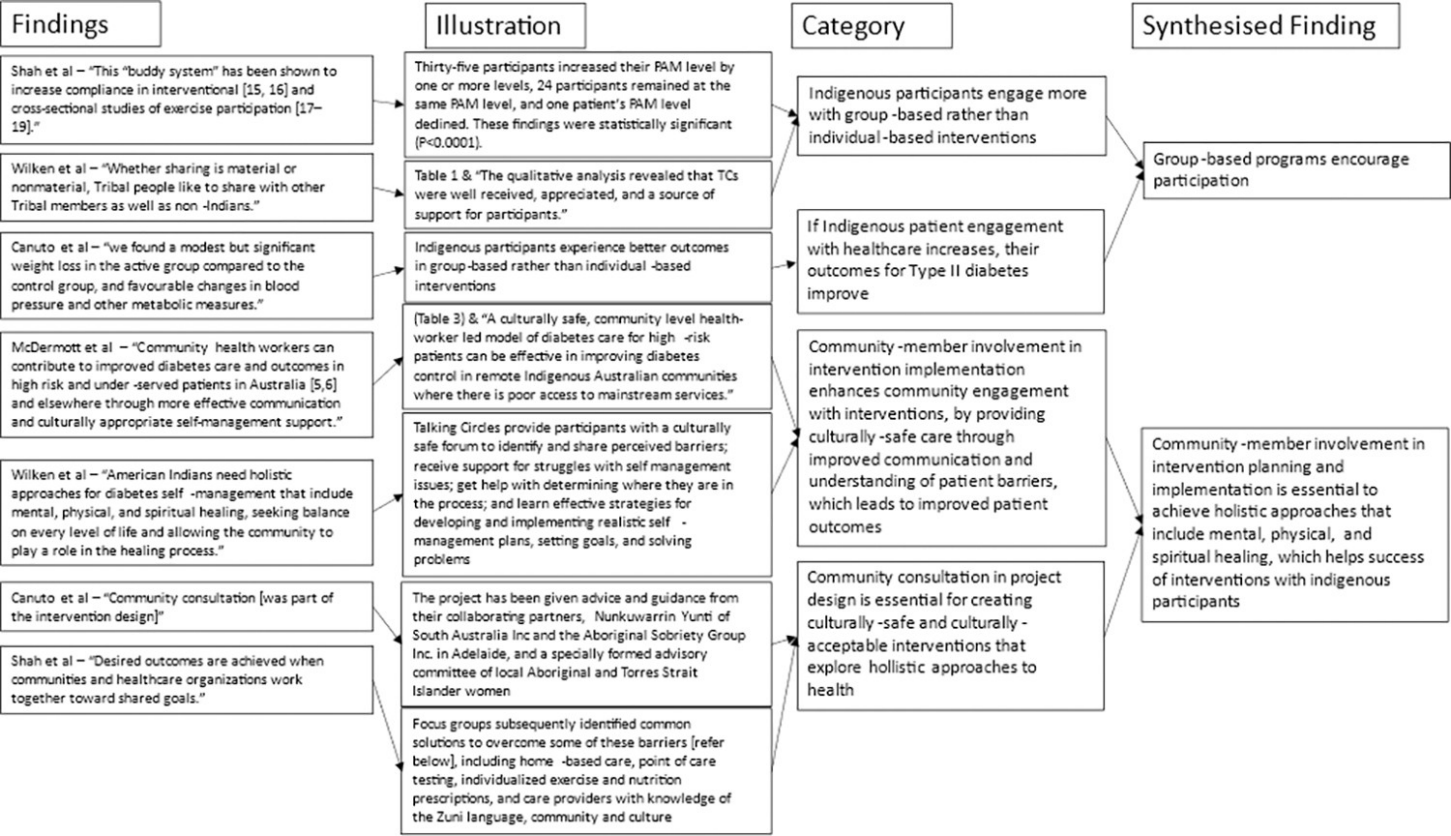
Flowchart of meta-aggregation part 3.

**Fig 5:**
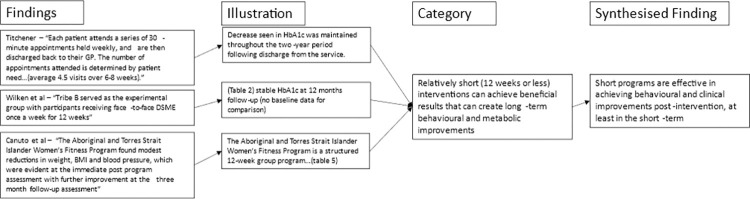
Flowchart of meta-aggregation part 4.

For the meta-aggregation of primary care design features, qualitative intervention aspects in each study associated with improvements in primary or secondary outcomes were extracted with direct illustrations from texts to support findings. Multiple findings were summarised into categories, which were then combined into synthesised findings that can inform policy [32, 33]. Critical appraisals were used to inform finding dependability [27]. The presence of illustrations (direct quotations or statistics) supporting text findings were used to inform confidence. If illustrations were present in all articles reporting a finding that finding was deemed unequivocal. If no illustrations were present for a finding it was deemed unsupported. Findings with any level of support in between were deemed equivocal. Furthermore, where the illustration supporting a finding was a statistically significant reduction in mean HbA1c or an illustration from a high-quality study, it was deemed unequivocal. This provided greater merit to studies with better quality and statistically significant results (Figs 2–5). Unequivocal findings of similar ideas or themes present in more than one article were combined into categories that were transformed into synthesised findings (Table 2). The review protocol was uploaded by PROSPERO on April 2, 2021 (registration: CRD42021240098).

**Table 2:** Summary of meta-aggregation findings.

SYNTHESISED FINDING	ARTICLES CONTAINING THIS FINDING (REFERENCED BY FIRST AUTHOR)	ARTICLES SUPPORTING FINDING WITH STATISTICALLY SIGNIFICANT HBA1C CHANGE	GOOD QUALITY STUDIES SUPPORTING FINDING
Indigenous or Community-based Health Workers yield improvements in clinical and behavioural parameters	McDermott [34]	McDermott [34]	McDermott [34]
	Pratte [35]	Shah [36]	Pratte [35]
	Shah [36]		
Policy and intervention designers must account for the dynamic interplay between health system barriers and community members’ ability to overcome these	McDermott [34]	McDermott [34]	McDermott [34]
	Smith [24]		Pratte [35]
	Pratte [35]		
	Canuto [37]		
Intuitively, when health systems barriers to primary care are removed, use of primary care increases	Smith [24]	Shah [36]	
	Shah [36]		
Empowering patients to self-manage their diabetes, through patient-centred education and workshop programs, effectively improves diabetes outcomes	Shah [36]	Shah [36]	
	Titchener [38]	Titchener [38]	
	Wilken [39]		
	Canuto [37]		
Group-based programs encourage participation	Shah [36]	Shah [36]	
	Wilken [39]		
	Canuto [37]		
Community-member involvement in intervention planning and implementation is essential	McDermott [34]	McDermott [34]	McDermott [34]
	Shah [36]	Shah [36]	
	Wilken [39]		
	Canuto [37]		
Short programs are effective in achieving behavioural and clinical improvements post-intervention, at least in the short-term	Titchener [38]	Titchener [38]	
	Wilken [39]		
	Canuto [37]		

## Results

Fig 1 details yields from the searches in the included databases using the search criteria. Of the 41 full-text articles screened, all texts were retrievable. 14 were removed due to a lack of reporting on HbA1c change. Another 7 were excluded based on study design or a lack of primary care intervention. A further 7 were excluded as they did not report separate outcomes for Indigenous participants. Another 2 studies included participants aged less than 18 years and were excluded. One study was excluded as it focused on South Asian ethnicity and another study was removed as it was an economic analysis of McDermott et al.’s 2015 study already included in the review. No studies were added for screening from grey literature. The remaining 7 studies were included for systematic review.

Three studies were cohort studies, two were quasi-experimental trials, and two were open label randomised control trials. Table 3 briefly summarises the intervention used in each study. Three studies were from the US, and two each were from New Zealand and Australia, none were from Canada. As per the inclusion criteria, and shown in Table 4, all studies reported change in mean HbA1c. Six included studies reported decreases in HbA1c, of which three studies had statistically significant results. However, no included studies reported diabetes-related hospitalisation rates. Five out of seven reported treatment adherence (of which three studies had statistically significant improvements), and six out of seven reported impact on mean weight/BMI (of which only McDermott et al.’s study showed a statistically significant improvement).

**Table 3:** Descriptive details of included studies.

Title	Author	Publication Year	Years covered	Study Design	Study location	Indigenous Population	Study Age	Study quality (overall JBI grade)	Primary care intervention	Control description (if applicable)	No. of Indigenous participants & Follow-up duration	Participants in intervention; control; total
Community health workers improve diabetes care in remote Australian indigenous communities: Results of a pragmatic cluster randomized controlled trial	McDermott et al.	2015	2011–2013	Open-label RCT	North Queensl-and	Aboriginal and/or Torres Strait Islander	18+, mean age 47.9 years	Good	Chronic care co-ordination from a community-based health worker at a primary care centre supported by a clinical outreach team.	Placed on waitlist (where intervention provided after 18 months)	213; Followed-up till 18-months post-intervention	100; 113; 213
Pragmatic randomised trial of a 12-week exercise and nutrition program for Aboriginal and Torres Strait Islander women: Clinical results immediate post and 3 months follow-up	Canuto et al.	2012	2010–2011	Open-label RCT	Adelaide, Australia	Aboriginal and/or Torres Strait Islander	18–64; intervention mean age 39.8 (95% CI 36.7–43.1); control 40.7 (37.7–43.6)	Moderate	The Aboriginal and Torres Strait Islander Women’s Fitness Program:	Placed on waitlist (offered structured 12-week exercise program after 12 months), provided same pedometer and exercise-diary, provided monthly newsletter with healthy recipes and exercise ideas, and invited to attend 4 1-hour dietician-facilitated workshops.	100; Followed-up till 3-months post-intervention	51; 49; 100
									12-week group program with			
									1) group exercise classes facilitated by qualified female fitness instructors			
									2) incidental activity education with free pedometer and exercise diary			
									3) 4 1-hour nutritional workshops delivered by female dieticians			
									4) positive reinforcement and encouragement through fortnightly newsletters with healthy recipes and exercise ideas as well as acknowledgment of the woman who completed most steps since previous newsletter			
Analysis of a primary care led diabetes annual review programme in a multi ethnic cohort in Wellington, New Zealand	Smith et al.	2011	2000–2006	Cohort Study	Wellington region, NZ	Maori	18+, mean age 63.6	Moderate	Free annual diabetes review (weight, height and, blood pressure, Laboratory tests: HbA1c, total and HDL cholesterol, triglycerides, and urinary microalbumin:creatinine ratio)	Not Applicable	298; Followed-up annually during 5 years of intervention but no post-intervention follow-up	Not Assessed
A patient-centred clinical approach to diabetes care assists long-term reduction in HbA1c	Titchener	2014	2008–2010	Cohort Study	New Zealand	Maori	18+	Poor	GPSI diabetes service	Not Applicable	29 (48 with 19 dropouts); Followed-up till 24-months post-intervention	Not Assessed
									1) weekly 30 minute appointment over 6–8 weeks then returned to GP.			
									2) Individualised diabetes management plan.			
									3) Individualised diabetes education			
Recruitment and effectiveness by cohort in a case management intervention among American Indians and Alaska Natives with diabetes	Pratte et al.	2019	2006–2008	Cohort Study	USA	American Indian and/or Alaskan Native	18+, majority 50+	Good	Chronic Care Model (Part of SDPI-HH):	Not Applicable	2910 (1123, 985, 802 in 2006, 2007, and 2008); Followed-up 12-months post-intervention	Not Assessed
									a) Case management (CVD risk assessment + individualised treatment plan)			
									b) individualised treatment plan			
									c) self-management education			
A home-based educational intervention improves patient activation measures and diabetes health indicators among Zuni Indians	Shah et al.	2015	2012	Quasi-experimental trial	New Mexico, USA	American Indian—Zuni	18+, mean 49.4	Moderate	6-month intervention featuring: 1) Monthly group lifestyle classes provided by Zuni community member health workers	Not Applicable	60; Followed to 6-months post-intervention	Not Assessed
									2) home-based individualised education and POCT			
									3) Advised to exercise 150min/week in culturally appropriate ways			
									4) peer support groups of 4–6 people			
									5) participant received $25 for each visit			
Talking Circles to Improve Diabetes Self-care Management	Wilken et al.	2017	Not provided	Quasi-experimental trial	United States	American Indian	21+	Moderate	DSME (Diabetes Self-Management Education with healthy lunch, diabetes education, healthy food + recipes) + TC after each DSME session (opened with prayer and smudging, run by tribal elder with diabetes, TC topics on living with diabetes and being strong in spirit) after each DSME + Nike Air 7 after completion	DSME (healthy lunch, diabetes education, healthy food + recipes) alone	39; Followed-up till 12-months post-intervention	20;19;39

**Table 4:** Summary of individual study impact estimates.

Title	Author	Publication Year	Impact on mean HbA1c	Diabetes-related hospitalisation rate	Impact on treatment adherance	Impact on mean weight/BMI
Community health workers improve diabetes care in remote Australian indigenous communities: Results of a pragmatic cluster randomized controlled trial	McDermott et al.	2015	−1.0% (intervention group: 10.8% to 9.8%) vs −0.2% (control group 10.6% to 10.3%) (p-value = 0.018)	Not Assessed	Adherence to all meds Intervention: 53% (95% CI 37.6–56.2) to 57% (43.7–62.8); Control 55% (45.1–64.9) to 41% (38.0–59.6)	Intervention: 89.7kg to 91.0kg; Control 91.4kg to 87.4kg control (72) -1.5kg (-2.7kg to -2.3kg 95% CI); intervention (71) -0.6kg (-2.0kg to 0.8kg)
Pragmatic randomised trial of a 12-week exercise and nutrition program for Aboriginal and Torres Strait Islander women: Clinical results immediate post and 3 months follow-up	Canuto et al.	2012	3 months follow up: Waitlist group: mean 6.6% (95% CI 5.9–7.2%) -> 6.4% (5.5–7.3%) Intervention group mean 6.2% (5.7–6.6%) -> 6.0% (5.4–6.6%)	Not Assessed	At 3 months follow-up Intervention group anthropometric follow up attendance: 53% vs 59% for control; Pathology follow-up intervention group 49% vs 47% for control	3 months follow up:WL group 94.8kg (95% CI 86.3–103.4) -> 79.2kg (73.3–85.0) Intervention group 92.6kg (84.5–100.6) -> 95.9kg (86.5–105.2) (influenced by loss to follow up)
Analysis of a primary care led diabetes annual review programme in a multi ethnic cohort in Wellington, New Zealand	Smith et al.	2011	Increased 0.03% (8.0% to 8.0%); linear term for curvilinear relationship was -0.05 (-0.10 to -0.015 95% CI)	Not Assessed	Oral hypoglycaemic use: (219/298) 73.5% -> (259/298) 86.9% (p < 0.001); Insulin use (31/298) 10.4% -> (56/298) 18.8% (p < 0.001); No insulin in participants with HbA1c >/ = 8% (106/129) 82.2% -> 87/126 (69.1%)	Mean change (Maori) -1.4kg 94.8 (SD 19.9) to 93.4 (SD 21.4) Linear term (95% CI -0.31 to -0.17) -0.24kg
A patient-centred clinical approach to diabetes care assists long-term reduction in HbA1c	Titchener	2014	(mmol/mol) Baseline = 100, Post-discharge:	Not Assessed	Not Assessed	Not Assessed
			3 months = 77 (p-value <0.001)			
			6 months = 77			
			9 months = 75			
			12–15 months = 87			
			18–24 months = 73			
			(no p-value for outcomes after 3-months follow-up)			
Recruitment and effectiveness by cohort in a case management intervention among American Indians and Alaska Natives with diabetes	Pratte et al.	2019	-0.14% (2006),	Not assessed	Increased healthy diet (p = 0.32) (0.05, 0.11, 0.10 increase in score 2006, 07, 08), decreased unhealthy diet (p = 0.75) (-0.15, -0.17, -0.13), increased physical activity (p = 0/0007) (0.43, 0.09, 0.07), decreased smoking (p = 0.37) (-3.9%, -2.9%, -3.1%)	BMI -0.21 (2006), -0.18 (2007), -0.16 (2008) p-value 0.93
			-0.18% (2007),			
			-0.31% (2008) (p-value = 0.17)			
A home-based educational intervention improves patient activation measures and diabetes health indicators among Zuni Indians	Shah et al.	2015	8.12 +- 2.16 —> 6 months follow-up 7.39 +- 1.6 (p-value = 0.001)	Not Assessed	Not Assessed	BMI 33.8 +- 8.4 —> 32.4 +- 8.2 (p = 0.001)
Talking Circles to Improve Diabetes Self-care Management	Wilken et al.	2017	No baseline data; (95% CI and P = 0.126) Intervention:	Not Assessed	At 3 months:Intervention group follow-up attendance 70% vs Control group 26.3% (p-value = 0.01)	Mean weight (lb) (95% CI) (p value 0.133) Intervention 3 months 211.5 (208.4–214.6), 6 months 210.1 (208.2–212.1), 9 months 208.0 (204.0–212.1), 12 months 206.7 (202.1–211.3); control 3 months 206.7 (203.9–209.4), 6 months 203.5 (198.7–208.4), 9 months 201.6 (196.4–206.8), 12 months 208.2 (197.7–218.6)
			3 months 8.69 (8.21–9.17),			
			6 months 9.02 (8.44–9.60),			
			9 months 8.85 (8.30–9.40),			
			12 months 8.72 (7.96–9.47)			
			Control:			
			3 months 9.00 (8.27–9.72),			
			6 months 9.83 (9.08–10.6),			
			9 months 9.62 (8.71–10.5),			
			12 months 9.62 (8.85–10.4)			

Using JBI checklists, three studies were determined as good quality, three moderate, and one study was poor quality [27] (Table 1). The first good quality study was McDermott et al.’s (2015) open-label RCT of chronic care co-ordination delivered by a community-based health worker supported by a clinical outreach team in Australia. Participants were follow-up 18 months post-intervention. Study groups were well-matched and despite greater loss to follow-up in intervention than control, authors achieved 90% power to detect mean HbA1c changes with their sample, providing internal validity [34]. Similarly, Canuto et al.’s (2012) RCT of an Australian 12-week small-group female exercise program with nutritional workshops, healthy information handouts, and free pedometer was a good quality study [37]. Study groups were followed 3 months post-intervention. Authors powered a sample of 13 to 80% to detect waist circumference change of 4cm and adequately accounted for loss to follow-up [40]. Pratte et al.’s (2019) cohort study of a chronic care model (Speical Diabetes Program for Indians–Healthy Heart Project (SDPI-HH)) in the US featuring cardiovascular risk assessment, care coordination, individual treatment programs, and diabetes self-management education was good quality [35]. Three separate cohorts were followed through a year of care coordination, with 12-months post-intervention follow-up. Although the study did not yield statistically significant HbA1c changes and lacks a control arm, authors accounted for the confounding role of dissimilar baseline health literacy and participation across cohorts. Appropriate statistical analysis of each cohort’s results also generated internal validity.

Smith et al.’s (2011) cohort study of free annual diabetes reviews featuring blood testing and counselling in NZ followed a cohort across 5 years but had no post-intervention follow-up [24]. Due to lack of control arm and inadequate analysis of loss to follow-up and confounding factors, the study was moderate quality. Both included quasi-experimental studies were of moderate quality. Shah et al.’s (2015) study of a 6-month program of monthly community-health worker lead group lifestyle classes, home-based education and testing, peer support, and $25 per session incentive for participation, is limited by lack of control arm [36]. Nevertheless, adequate statistical analysis powered HbA1c findings for its sample of 60, providing some internal validity. Wilken et al.’s (2017) study of a diabetes self-management education program with lifestyle education, talking circles lead by community elders, and free Nike Air 7’s as incentive for participation, featured a well-matched control arm [38]. However, there was greater loss to follow up in the control arm, which was not statistically encountered for, generating selection bias.

Finally, Titchener’s (2014) poor quality cohort study of a 6–8 week program with weekly GP appointments featuring individualised diabetes management plans and self-management education was included [38]. The study did not feature a control arm and did not account for the impact of selection bias. Māori participants experienced disproportionate dropout and authors did not comment on sample size for powering HbA1c findings. S2 Table provides more detailed study precis.

Seven common elements with high dependability and unequivocal confidence were identified among various primary care interventions in included studies. Four studies demonstrated community consultation is essential to achieve holistic approaches that include mental, physical, and spiritual healing to facilitate uptake and therefore improved outcomes of interventions with Indigenous participants. Three interventions demonstrated success of community health worker (CHW)-led case management and meaningful patient education in improving patient engagement with diabetes care. Four studies demonstrated that the dynamic interplay between health system barriers and community members’ ability to overcome these must be accounted for in intervention and primary care service design. In turn, two studies demonstrated when healthcare providers reduced barriers to healthcare access and use, Indigenous people increased engagement with healthcare and consequently their clinical parameters improve. Similarly, four studies featuring empowering Indigenous participants, through self-management and diabetes education, showcased improved engagement with healthcare and improved clinical outcomes. Three studies also demonstrated potential benefits of short intensive programs (12 weeks or less) and group-based programs.

## Discussion

This study pooled quantitative findings and qualitative factors from seven studies of primary care interventions managing T2DM in First Nations adults from high income countries. This builds on a previous study which sought to analyse primary care attributes for T2DM management of Indigenous populations, but had insufficient data to form conclusions for practice [12]. Our study analyses an additional decade of literature in this field where research is limited. Our findings are therefore vital for informing evidence-based policy decisions [41, 42]. Seven unequivocal synthesised findings were found by meta-aggregation. These seven features may be translated to different Indigenous populations living in high income countries to enhance acceptance of care and improve clinical outcomes.

Findings are presented as a list to display the yields of the statistical approach used for this study. However, in the real-world, these findings are all interrelated. Ultimately, in line with the study aim, all findings work to reduce and resolve health system barriers (finding 2) pushing Indigenous people away from participating in the health system. CHWs (finding 1) reduce health system barriers against primary care access (finding 2) through their role in case management. CHWs further reduce health system barriers by reassuring patients that the health system will understand them, rather than making assumptions based on untrue stereotypes [43, 44]. Finding 3 also works to negate health system barriers (finding 2) by empowering Indigenous people to self-manage their diabetes and not rely on the health system. Moreover, CHWs (finding 1) are proven to be effective in patient education in self-management [34, 36, 39, 45]. Additionally, CHWs (finding 1) are inherently a form of community-member involvement in intervention implementation (finding 4). Findings 1–4 have greater importance and, along with finding 5, have breadth to be applied across multiple settings. Contrarily, findings 6 and 7 are elements that can be applied to specific interventions.

### 1. Indigenous or community-based health workers yield improvements in clinical and behavioural parameters

McDermott et al. and Shah et al. demonstrated statistically significant HbA1c decreases using CHWs to case manage individual patient care by facilitating engagement with appointments and providing education in ways that are meaningful to patients [34, 36]. In remote communities in geographically challenging areas, CHWs may assist transporting patients to primary care appointments or fresh grocery centres, further reducing barriers to their diabetes management [34, 36, 39]. McDermott et al. demonstrated improvements in some health behaviours (dietician referral and blood glucose self-monitoring) in intervention group, but deterioration in others (smoking and dyslipidaemia), making it difficult to correlate Indigenous Health Workers to health behaviours in this study. A recent qualitative analysis of Aboriginal and Torres Strait Islander health services found staff and community members valued Aboriginal and Torres Strait Islander team members in delivering care [44]. Pratte et al. also demonstrated an improvement in clinical and behavioural outcomes with case management being a main component of intervention [35].

### 2. Intuitively, when health systems barriers to primary care are removed, use of primary care increases

Two studies displayed that removing barriers to healthcare (through free check-ups, trusted staff members, and material incentives) enhanced participation in interventions, which in turn improved clinical outcomes [25, 36]. Of these, the intervention in Shah et al.’s article, which featured at-home individualised education, achieved statistically significant HbA1c decrease. Likewise, a well-designed Canadian study, excluded from review due to lack of Indigenous-specific results, featured diabetes management plans delivered directly at homeless shelters for homeless people living with DM2 without a fixed address or contact information for clinic appointment details [45]. In keeping with synthesised finding 4, community consultation was a major strength of the intervention, enabling adaptations of intervention design based on target population socioeconomic circumstances. The study demonstrated 1.1% (p <0.05) reduction in HbA1c and 50% increase in blood sugar self-monitoring (p<0.01) in 15 homeless people who were eligible and attended all follow-up [45]. This further displays the benefit of reducing access barriers in improving clinical outcomes for disadvantaged populations.

### 3. Empowering patients to self-manage their diabetes, through patient-centred education and workshop programs, effectively improves diabetes outcomes

Four studies demonstrated empowering patients by use of patient-centred approaches to enhance understanding of diabetes, treatments, and self-management, improved clinical parameters [34, 35, 37, 39]. Of these, Shah et al. and Titchener et al. both used individualised education and care plans to achieve statistically significant HbA1c reductions; Shah et al. also used home-based education sessions as a method to reduce health system barriers to reduce healthcare, linking in with the second finding [36, 38]. Utilising longer follow-up periods in real-world studies of First Nations populations may demonstrate the benefit of patient empowerment in creating sustained clinical and behavioural change, as has been displayed in other populations [46].

### 4. Community member involvement in intervention planning and/or implementation is essential

The same four studies, including McDermott et al. and Shah et al., featured community involvement in intervention planning and implementation to achieve a holistic approach with mental, physical, and spiritual healing, which engaged Indigenous participants. This adds to the extensive evidence and government and Indigenous leadership advocacy for Indigenous leadership in research, community-based problem definitions, community-based participatory research, and Indigenous ontological, epistemological, methodological, and axiological integration into health research and practice [14, 17, 21, 25, 47, 48]. An Alaska-based community-owned Indigenous Health Service study (excluded from review for lack of HbA1c reporting) modelled the impacts of multiple simultaneous changes to primary care delivery, including use of physician-lead integrated care teams, improved access, and case management. These changes were introduced after focus groups with local Alaska Natives or American Indians. Authors demonstrated a non-statistically significant increase (1.69%, p = 0.658) in uptake of annual HbA1c screening a decade after implementation [15].

Another study, excluded for not reporting HbA1c, surveyed physicians who participated in workshops on patient-centred approaches to diabetes management and education for Indigenous patients in Canada [49]. The workshop was designed using Indigenous-based problem definition arising from focus groups with Indigenous patients and health educators. Physicians reported improved understanding of psychosocial and cultural factors affecting Indigenous patients with diabetes and felt more confident to adapt services to deliver patient-centred care [49]. This demonstrates the benefits of Indigenous partnership in facilitating primary care design accepted by Indigenous patients. This is supported by a 2015 systematic review of Indigenous people’s participation in RCTs in Australia, Canada, NZ, and the US. The review concluded relationship and partnership building with Indigenous communities and drawing on Indigenous knowledge models in intervention design were key facilitators to Indigenous acceptability of interventions [50].

### 5. Policy and intervention designers must account for the dynamic interplay between health system barriers and community members’ ability to overcome these

In the real-world, health system barriers can change. During the COVID-19 pandemic, additional barriers such as limited occupants in waiting rooms, arose as new health system barriers [14, 18]. Similarly, Indigenous community factors, such as community funerals or occupation seasons and its impact on income or job rosters, can reduce availability for health appointments [1, 51]. Effective primary care must have capacity to adapt to these natural changes. Four reviewed studies showcased significant variation in intervention impact with time across study cohorts or follow-up [24, 34, 35, 37]. Interventions must account for the dynamic nature of the target population and its willingness to stay connected to interventions. Furthermore, the driving factors behind changes in levels of engagement with interventions must be more clearly examined. Mid-intervention engagement strategies, such as new material incentives or changes to place of intervention delivery, may be considered in Indigenous health research to maintain participation. Wilken et al.’s talking circle pilot study successfully increased control group participation in post-intervention follow up by using a small incentive at six-months follow-up [39].

### 6. Short programs are effective in achieving behavioural and clinical improvements post-intervention, at least in the short-term

Three studies demonstrated intensive 12-week programs can achieve sustained change at 3-12-month follow up [37–39]. Of these, Titchener’s study achieved statistically significant HbA1c decrease at three months [38]. This is possibly due to shorter programs maintaining participation for the entire duration of intervention. This strategy may alleviate the discussed dynamic nature of barriers against health system access. Longer follow-up periods in studies are required to enable evaluation of success of such interventions to create sustained change in outcomes and behaviours.

### 7. Group-based programs encourage participation

The use of group-based interventions in three included studies warrants its mention as a separate synthesised finding [36, 37, 39]. Furthermore, the three studies reported favourable reactions by Indigenous groups towards the intervention. Only one study achieved statistically significant HbA1c reduction [36], and Wilken et al.’s good quality study showed statistically significant increased engagement with management in intervention group compared to control [39]. This finding accounts for the overly individualistic nature of western healthcare, which is a sheer contrast to the value of wellbeing as a community manifestation held by many Indigenous groups. The individualistic aspect of western healthcare can act as a barrier to facilitating optimal use of the health system. Group-based interventions can dismantle this barrier (synthesised finding 2) and encourage Indigenous participants to improve their own health outcomes and help fellow community members achieve the same simultaneously.

### Strengths–Adequate analysis for recommendations

The robust protocol and inclusion of only studies reporting results separately for Indigenous participants ensures relevance to the target populations’ shared experiences. The use of mean HbA1c reporting as an inclusion criterion significantly limited the number of eligible studies. However, this indicator of glycaemic management allows for relatively standardised outcome comparisons between disparate study designs [28]. The mixed-method analysis allows reliable qualitative lessons to be extracted despite variance in quantitative reporting [32, 33].

### Strengths–External validity of findings

Given the variation in quality of studies, it is difficult to interpret quantitative findings. McDermott et al.’s study was the only good quality study with statistically significant HbA1c change [34]. Authors did not explore the role of intervention bias for the intervention group, but this is unlikely to account for the magnitude of HbA1c change in intervention. Samples had similar demographics to other Indigenous populations with poorly-controlled diabetes in primary care (majority (66.4%) female, and high burden of obesity, smoking, and unemployment (52.2%)), making results externally valid [34]. In Pratte et al.’s study, at baseline cohorts also had similar demographics to other Indigenous groups attending primary care with diabetes (61.2–69% female, and high burden of unemployment (26–32.3%) and obesity), rendering findings externally valid [35].

### Limitations–Challenges of conducting indigenous health research

The review’s limitations may reduce transferability of findings across Indigenous populations in high income countries. No articles from Canada were eligible under our selection criteria. This may reflect the use of English language as a selection criterion or a lack of T2DM research with specific HbA1c reporting in Canada. Furthermore, many limitations stemmed from included studies being observational and moderate-to-low quality with significant loss to follow-up. Participants engaging with interventions and follow-up are generally more motivated with superior outcomes, rendering studies prone to selection bias. Many included studies lacked probability sampling and were prone to sample bias [24, 35, 38, 39]. All included studies featured short post-intervention follow-up (<2 years). This limits evaluation of intervention and impact sustainability. These limitations are a by-product of the challenges in performing appropriate Indigenous research. Distrust among Indigenous groups towards health research, hesitancy of Indigenous groups to accept protocols featuring no-intervention control groups, and complexity of analysing the impact of social determinants of health reduces engagement by researchers and participants alike [1, 34, 37, 49, 50]. These challenges cause variance in study design and reporting, making summary measures difficult to produce [32, 33].

### Limitations–Inconsistencies in outcome reporting dictated broad selection of secondary outcomes

During initial review of Indigenous T2DM research, inconsistencies in outcome reporting were observed. Consequently, a broad range of qualitative and quantitative indicators were selected as secondary outcomes for this systematic review. As displayed in Table 4, included studies used varied indicators for secondary outcomes. For example, McDermott et al. used mean change in weight (kg) as a measure of impact on weight/BMI, whereas Pratte et al. used mean change in BMI [34, 35]. Moreover, due to the nature of interventions, proxy measures used for treatment adherence varied between studies. Smith et al. reported changes in oral hypoglycaemic agent use, whereas Pratte et al. reported changes in smoking rates and healthy diet [25, 35]. Such a broad selection of secondary outcomes enabled extraction of greater amounts of data from eligible studies but reduced the comparability of secondary outcomes.

### Recommendations for future research–Improved scientific rigour whilst maintaining community wellbeing and partnership

To overcome barriers to Indigenous research, researchers trusted by First Nations’ communities should advocate for greater scientific rigour when consulting communities in intervention planning, whilst maintaining community safety [32, 33]. Additionally, non-Indigenous researchers should improve research protocols to facilitate Indigenous group involvement in establishing terms of conduct for research affecting them and promote Indigenous participation as equal partners [51]. Specifically for Indigenous T2DM research, guidelines recommending at least two-year post-intervention follow-up, powering of sample size, and standardisation of outcome reporting should be generated. There is also need for more studies using HbA1 reporting to evaluate primary care interventions in Canada’s First Nations people.

Scientific rigour should not come at the cost of community wellbeing. One-sided partnerships dominated by researchers in community-based research have contributed to distrust of western medicine systems by Indigenous groups [25]. Advocating for study designs which maintain community safety, the paramount priority in community-based research, may compromise true scientific methods. For example, the use of waitlist for control rather than a non-intervention control, seen in two of the included studies [34, 37], compromises long-term comparison data but ensures equal access to potentially beneficial interventions. Future researchers must maintain trust of Indigenous groups through their work.

### Recommendations for future research–Integrating primary care surveillance systems with hospital admission date

No included studies reported hospitalisation rates during intervention. Diabetes is an independent risk factor for hospitalisation and increased hospitalisation duration [52, 53]. More primary care centres should track hospitalisation rates of diabetic patients to monitor the impact of their care design on hospitalisation.

### Recommendations for future research–Accounting for pharmacological effects on body weight and participant baseline body weight

Weight can be difficult to interpret in interventions involving pharmacological diabetes treatment. McDermott et al. and Canuto et al. found non-statistically significant weight gain in intervention groups but weight loss in control groups [34, 37]. Although weight loss is a non-pharmacological management strategy for diabetes, some hypoglycaemic medication classes cause weight gain [54]. Therefore, medication adherence can confound weight gain. Additionally, several studies experienced significant loss-to follow up and used intention-to-treat analysis [34, 36–38]. This does not account for baseline demographics. If outliers in body mass were lost to follow-up or remained in the study the mean is skewed, which can significantly alter results.

### Recommendations for future research–Longer post-intervention follow-up

Treatment adherence results were difficult to interpret. Intervention groups in studies with control arms displayed greater attendance for follow-up, which is likely intervention bias [34, 37, 39]. The intervention group in McDermott et al.’s study displayed a decrease in all medication adherence at the end of the intervention, contrarily control group had increased all medication adherence [34]. Titchener’s post-intervention follow-up of 24-months was the longest in included studies, though p-values were not calculated for results after three-months [38]. Future study designs may benefit from longer post-intervention follow-up given chronic disease management requires long-term adherence. This is well-demonstrated in Trevisi et al.’s quasi-experimental study, which followed up patients with diabetes in Navajo Nation who received Community Health Representative-led case management and health education as part of the Community Outreach and Patient Empowerment (COPE) project [55]. The study was not found in the librarian-supervised keyword search for this study, as the intervention was not based in primary care. The study followed-up behavioural and clinical outcomes 24-months post-CHW intervention and found statistically significant sustained reductions in HbA1c after 24-months in patients who received CHW compared to patients who did not.

## Conclusions

This study analyses a decade of research in a field with disproportionate burden of disease and limited research. Indigenous group engagement with chronic disease management, including T2DM, is challenged by the ongoing impacts of colonialism, socioeconomic hardship, and racism. High income economies have relatively large pools of resources for their health systems that need more effective application to reduce barriers preventing healthcare access and use for Indigenous communities, given the ongoing disproportionate burden of disease. There is room for more robust studies with Indigenous populations that maintain community wellbeing and will aid the reliability of future analyses. This review attempts to identify and evaluate interventions to enable healthcare providers to reduce health system barriers against access and enhance Indigenous participant engagement with T2DM management to improve clinical outcomes. Study interventions featuring CHWs, consideration of dynamic nature of health system barriers and community member availability, a focus on patient empowerment, and community-member involvement in planning and implementation performed favourably in reducing health system and primary care barriers. This engaged Indigenous patients and improved clinical and behavioural outcomes. These attributes should be given priority in primary care policies for the management of T2DM in Indigenous adult populations in high income countries. Our findings may assist decision-making for policy makers designing interventions for management of chronic diseases in Indigenous populations to close the gap in health disparities globally.

## Supporting information

S1 Table Search strategy. (DOCX)

S2 Table Included studies precis table. (DOCX)

S1 Fig JBI critical appraisal tools. (DOCX)
