# Supplementary material for: Effective primary care management of type 2 diabetes for indigenous populations: A systematic review
Source: PLoS One. 2022 Nov 10;17(11):e0276396. doi: 10.1371/journal.pone.0276396 (PMC9648771; doi:10.1371/journal.pone.0276396)
Supplement: S1 Table — (DOCX) [file pone.0276396.s001.docx]

Appendix 1 - Search Strategy

| **Search Identification Number** | **Search Terms** |
| --- | --- |
|  |  |
| 1 | "primary care" OR "health care" OR "healthcare" OR "patient care" OR "patient cares" OR "Health Services Administration" OR "Health Priorities" OR "Health Resource Allocation" OR "Health Resource Utilisation" OR "Health Service Accessibility" OR "Right to Health" OR "Managed Care Programs" OR "primary medical care" OR "first line care" OR "primary care nursing" OR "primary nursing care" OR "health maintenance organisation" OR "Independent practice Association" OR "ACCHS" OR "ACCHO" OR "NACCHS" OR "NACCHO" OR "Aboriginal Community Controlled" OR "intersectoral cooperations" OR "Health maintenance Organisations" OR "Independent practice Associations" OR "National health Programs" OR "national health program" OR "Telehealth" OR "Telemedicine" OR "Telenursing" OR "Telenutriton" OR "Medicare" OR "Medicaid" OR "health planning" OR " health and welfare planning" OR "State Medicine" OR "intersectoral collaboration" OR "intersectoral collaborations" OR "intersectoral cooperation" OR "eligibility determination" OR "health plan implementation" OR "health plan implementations" OR "Capacity building" OR "national health insurance" OR "national health service" OR "national health services" OR "clinical governance" OR "fee schedule" OR "fee schedules" OR "health facility administration" OR "Management audit" OR "management audits" OR "operational audit" OR "operational audits" OR "multi hospital system" OR "multi institutional system" OR "multi-hospital system" OR "multi-institutional system" OR "multihospital system" OR "shared hospital service" OR "shared hospital services" OR "hospital shared service" OR "hospital shared services" OR "organizational change" OR "organizational changes" OR "organizational innovation" OR "organizational innovations" OR "voluntary programs" OR "voluntary program" OR "case management" OR "managed care programs" OR "care continuity" OR "care continuum" OR "continuity of care" OR "continuity of patient care" OR "continuum of care" OR "patient care continuity" OR "medical home" OR "medical homes" OR "patient centered care" OR "patient centered nursing" OR "patient focused care" OR "patient-centered care" OR "patient-centered nursing" OR "patient-focused care" OR "patient centred care" OR "patient-centred care" OR "transition care" OR "transition cares" OR "transitional care" OR "transitional cares" OR "patient navigation" OR "patient navigations" OR "patient navigator" OR "patient navigators" OR "clinical path" OR "clinical paths" OR "clinical pathway" OR "clinical pathways" OR "cultural care" OR "culturally competent care" OR "culturally competent health care" OR "culturally congruent care" OR "access to health care" OR "accessibility of health services" OR "availability of health services" OR "health services accessibility" OR "health services availability" OR "health services geographic accessibility" OR "program accessibility" OR "clinical practice pattern" OR "clinical practice patterns" OR "practice gap" OR "practice gaps" OR "professional practice gap" OR "professional practice gaps" OR "Health equity" OR "indigent care" OR "uncompensated care" OR "Disease management" OR "Disease managements" OR "medication reconciliation" OR "medication reconciliations" OR "drug therapy management" OR "medication therapy management" OR "point of care" OR "health care team" OR "health care teams" OR "interdisciplinary health team" OR "interdisciplinary health teams" OR "medical care team" OR "medical care teams" OR "patient care team" OR "patient care teams" OR "Learning health system" OR "Learning Health Community" OR "Health priorities" OR "Health Resource Allocation" OR "Health Care Rationing" OR "Health Resource Allocations" OR "Rationing of Health Care" OR "Health Resource Utilisation" OR "Health Resources Utilisation" OR "Health Resources Utilization" OR "Health Service Purchasing" OR "Health Services Purchasing" OR "HMO" OR "Health Maintenance Organisations" OR "Health Maintenance Organization" OR "Prepaid Group Health Organization" OR "Prepaid Group Health Organizations" OR "Independent Physicians Association" OR "Independent Practice Association" OR "Independent Practitioners Association" OR "Independent Provider Association" OR "Individual Practice Association" OR "Safety-Net Providers" OR "Safety-Net Clinics" OR "Safety-Net Hospitals" OR "Mobile Health" OR "eHealth" OR "mHealth" OR "Remote Consultation" OR "Consultations Remote" OR "Remote Consultations" OR "Teleconsultation" OR "Teleconsultations" OR "health indigenous service" OR "health indigenous services" OR "Indigenous health service" OR "indigenous health services" OR "Traditional Healer" OR "Indigenous Healer" OR "Indigenous Healers" OR "Medicine Men" OR "Traditional Healer" OR "Witch Doctor" OR "Witch Doctors" |
| 2 | "adult-onset diabetes mellitus" OR "ketosis-resistant diabetes mellitus" OR mody OR "maturity onset diabetes" OR "maturity-onset diabetes" OR "maturity-onset diabetes mellitus" OR niddm OR "non-insulin-dependent diabetes mellitus" OR "noninsulin dependent diabetes mellitus" OR "noninsulin-dependent diabetes mellitus" OR "slow-onset diabetes mellitus" OR "stable diabetes mellitus" OR "type 2 diabetes" OR "type 2 diabetes mellitus" OR "lipoatrophic diabete" OR "lipoatrophic diabetes" OR "Ketosis-Resistant Diabetes Mellitus" OR "Maturity-Onset Diabetes Mellitus" OR "NIDDM" OR "Non-Insulin-Dependent Diabetes Mellitus" OR "Slow-Onset Diabetes Mellitus" OR "Stable Diabetes Mellitus" OR "Type 2 Diabetes" OR "Type 2 Diabetes Mellitus" OR "Type II Diabetes" OR "Type II Diabetes Mellitus" OR "Prediabetes" OR "Prediabetic state" OR "type two diabetes" OR "type two diabetes mellitus" |
| 3 | "first nation" OR "first nations" OR "indigenous" OR "native" OR "natives" OR "inuit" OR "inuits" OR "aborigine" OR "aborigines" OR "australian race" OR "australoid race" OR "australoid races" OR "oceanic ancestry group" OR "oceanic ancestry groups" OR "pacific island american" OR "pacific island americans" OR "pacific islander american" OR "pacific islander americans" OR "Canadian Indian" OR "aleut" OR "nuit" OR "pima" OR "cree" OR "cherokee" OR "American native continental ancestry group" OR "central American" OR "north American" OR "South American" OR "eskimos" OR "eskimo" OR "Maori" OR "Aboriginal" OR "Torres Strait Islanders" OR "Torres Strait Islander" OR "American Indian" OR "American Indians" OR "Torres Strait" OR "South Sea Islander" OR "South Sea Islanders" |
| 4 | 1 AND 2 AND 3 |
