# Supplementary material for: Effective primary care management of type 2 diabetes for indigenous populations: A systematic review
Source: PLoS One. 2022 Nov 10;17(11):e0276396. doi: 10.1371/journal.pone.0276396 (PMC9648771; doi:10.1371/journal.pone.0276396)
Supplement: S2 Table — (DOCX) [file pone.0276396.s002.docx]

Appendix 3 - Included Studies Precis Table

| Article | Precis |
| --- | --- |
| **Community health workers improve diabetes care in remote Australian indigenous communities: Results of a pragmatic cluster randomized controlled trial (2015)^(34)^** | McDermott et al’s open-label randomised controlled trial (RCT) in an Aboriginal and/or Torres Strait Islander group from North Queensland, Australia, demonstrated a greater decrease in HbA1c in the intervention group (−1.0% (10.8% to 9.8%)) compared with the control group (−0.2% (10.6% to 10.3%)) (p-value = 0.02). The intervention group (100) received care coordination by a community-based health worker, while the control group (113) was placed on an 18-month waitlist to receive care coordination. The intervention group demonstrated an increase in medication adherence (53% (95% CI 37.6-56.2) to 57% (43.7-62.8)) unlike the control group (55% (45.1-64.9) to 41% (38.0-59.6)). However, the intervention group experienced less weight loss (control -1.5kg (-2.7kg to -2.3kg 95% CI); intervention -0.6kg (-2.0kg to 0.8kg)). |
| **Pragmatic randomised trial of a 12-week exercise and nutrition program for Aboriginal and Torres Strait Islander women: Clinical results immediate post and 3 months follow-up (2012)^(40)^** | Canuto et al’s open-label RCT involved Aboriginal and/or Torres Strait Islander people from Adelaide, Australia, in which 51 received the intervention (12-week group exercise program with nutrition and lifestyle education) and 49 received the control (nutrition and lifestyle education only). At 3 months follow-up, control group HbA1c decreased from 6.6% (95% CI 5.9-7.2%) to 6.4% (5.5-7.3%), whereas in the intervention group HbA1c decreased from 6.2% (5.7-6.6%) to 6.0% (5.4-6.6%). Follow-up attendance showed mixed results for intervention and control group. The control group experienced weight loss (94.8kg (95% CI 86.3-103.4) to 79.2 (73.3-85.0)), whereas the intervention group experienced weight gain (92.6kg (84.5-100.6) to 95.9kg (86.5-105.2). |
| **Analysis of a primary care led diabetes annual review programme in a multi ethnic cohort in Wellington, New Zealand (2011)^(24)^** | Smith et al’s cohort study from Wellington, New Zealand, described the results of an audit of 5 years of patient data following government provision of free annual health checks for adults with diabetes under the Get Checked program. Of the 298 Māori participants who attended a check up each of the 5 years, HbA1c increased 0.03% (8.0% to 8.0%), which was demonstrated to be an annual reduction of 0.05% (-0.10 to -0.015 95% CI) when accounting for the natural history of rise in HbA1c with time. Oral hypoglycaemic use and insulin use increased whilst participants with HbA1c <8% not on insulin decreased. Furthermore, Māori participants demonstrated a mean weight loss of 0.24kg (95% CI -0.31 to -0.17) each study year. |
| **A patient-centred clinical approach to diabetes care assists long-term reduction in HbA1c (2014)^(36)^** | Titchener’s cohort study of 48 Māori people in New Zealand saw a change in HbA1c from 100 mmol/mol at baseline to 77 at 3 months post-discharge (p-value <0.001). The 6-8 week intervention featured 30-minute appointments with a GP with special interest in diabetes providing individualised diabetes management plans and education. The study did not report on any of the secondary outcomes. |
| **Recruitment and effectiveness by cohort in a case management intervention among American Indians and Alaska Natives with diabetes (2019)^(37)^** | Pratte et al’s Cohort Study of American Indian and/or Alaskan Native people in USA reported analysis from the chronic care model institutionalised through the Special Diabetes Program for Indians Healthy Heart (SDPI-HH) project. The intervention featured individualised risk assessment, treatment, and self-management education. 2910 participants were included across three years of audit data. Each cohort (2006-08) experienced non-significant HbA1c decrease and weight loss. Authors report participants showed increased healthy diet uptake, decreased unhealthy diet, and decreased smoking. All cohorts reported non-significant reductions in BMI. |
| **A home-based educational intervention improves patient activation measures and diabetes health indicators among Zuni Indians (2015)^(38)^** | Shah et al’s quasi-experimental trial saw a combination of five diabetes-targeted lifestyle modification interventions, including monthly lifestyle classes delivered by Zuni community health workers, provided to a group of 60 Zuni American Indians across 6 months in New Mexico, US, in 2012. The study reported a decrease in HbA1c from 8.12% +- 2.16% to 7.39% +- 1.6% (p-value 0.001) at the end of the intervention. The study did not report on treatment adherence, but reported a decrease in mean BMI from 33.8 +- 8.4 to 32.4 +- 8.2 (p-value 0.001). |
| **Talking Circles to Improve Diabetes Self-care Management (2017)^(39)^** | Wilken et al’s quasi-experimental study of 39 American Indians in the US, saw 20 receive the intervention of diabetes self-management education followed by diabetes talking circles lead by community elders. 19 people received the control of diabetes self-management education alone. Non-significant HbA1c changes occurred in intervention and control groups. At 3 months, adherence to follow-up was greater in the intervention than control group (70% vs 26.3% (p-value = 0.01)). Non-significant weight loss was reported in both groups. |
